# First Insights into the Fecal Metabolome of Healthy, Free-Roaming Giraffes (*Giraffa camelopardalis*): An Untargeted GCxGC/TOF-MS Metabolomics Study

**DOI:** 10.3390/metabo14110586

**Published:** 2024-10-28

**Authors:** Andri Grobbelaar, Gernot Osthoff, Ilse du Preez, Francois Deacon

**Affiliations:** 1 Department of Animal Sciences, Faculty of Natural and Agricultural Sciences, University of the Free State, P.O. Box 339, Bloemfontein 9300, South Africa; andri.giraffe@gmail.com; 2 Department of Microbiology and Biochemistry, Faculty of Natural and Agricultural Sciences, University of the Free State, P.O. Box 339, Bloemfontein 9300, South Africa; osthoffg@ufs.ac.za; 3 Centre for Human Metabolomics, North-West University, Potchefstroom 2531, South Africa; ilse.dupreez@nwu.ac.za

**Keywords:** feces, giraffe, metabolites, pesticides, nutrition, ruminant, season, sex

## Abstract

Background/Objectives: This study provides the first insights to the fecal metabolome of the giraffe (*Giraffa camelopardalis*). By using untargeted metabolomics via gas chromatography time-of-flight mass spectrometry (GCxGC/TOF-MS), this study primarily aims to provide results of the impact that external stimuli, such as supplemental feeding (SF) practices, seasonal variation and sex, might have on the fecal metabolome composition of healthy, free-roaming giraffes. Methods: Untargeted GCxGC/TOF-MS analysis was applied to the feces collected from thirteen giraffes (six males and seven females) from six different locations within the central Free State Province of South Africa over a period of two years. Statistical analysis of the generated data was used to identify the metabolites that were significantly different between the giraffes located in environments that provided SF and others where the giraffes only fed on the natural available vegetation. The same metabolomics analysis was used to investigate metabolite concentrations that were significantly different between the wet and dry seasons for a single giraffe male provided with SF over the two-year period, as well as for age and sex differences. Results: A total of 2042 features were detected from 26 giraffe fecal samples. Clear variations between fecal metabolome profiles were confirmed, with higher levels of amino acid-related and carbohydrate-related metabolites for giraffes receiving SF. In addition, a separation between the obtained profiles of samples collected from a single adult male giraffe during the wet and dry seasons was identified. Differences, such as higher levels of carbohydrate-related metabolites and organic compounds during the wet season were noted. Distinct variations in profiles were also identified for the metabolites from fecal samples collected from the six males and seven females, with higher concentrations in carbohydrate-related metabolites and alkanes for female giraffes comparatively. Conclusions: This is the first study to investigate the composition of the fecal metabolome of free-roaming giraffes, as well as the effects that external factors, such as environmental exposures, feeding practices, seasonal variations, age and sex, have on it. This novel use of fecal metabolomics assists in developing non-invasive techniques to determine giraffe populations’ health that do not require additional stressors such as capture, restraint and blood collection. Ultimately, such non-invasive advances are beneficial towards the conservation of wildlife species on a larger scale.

## 1. Introduction

The study of the key roles that gut microbiota and their associated metabolic processes have on host food digestion, energy supplementation, health and overall metabolism has become more evident in the last decade [1,2]. Fecal metabolites are effectively the end products of the complex digestive processes occurring inside the animal and events, exposures or phenomena occurring outside the animal (the environment). Feces are directly associated with the digestive system and health status of an individual and may reflect changes in the mammalian host’s diet and metabolism very early [3,4,5].

In comparison to human and crop health, the application of metabolomics to livestock research is, however, somewhat less widely used or appreciated [6]. Even less is known about the benefits that metabolomics research could have in the wildlife industry and conservation. The comprehensive measurement of metabolites (via metabolomics) could, however, provide better insights to determine the specific interactions between a biological specimen and the internal fluctuations and/or external disturbances to which it is exposed [7,8]. The systematic study of the small compounds left behind by specific cellular processes helps us to better understand the metabolic processes defining the phenotype of biological samples [3,9]. By using highly sensitive analytical equipment and complex statistical procedures, metabolomics provides a non-biased snapshot of all the detectable metabolites in a biological system [4,10,11]. The integrated impact of internal (e.g., genetics) and external (e.g., environment) stimuli on metabolism can also be defined by the study of composition, dynamics, relative abundance and interactions among metabolites in a particular organism [10,12].

Invasive methods, such as blood collections and necropsies, which are used to determine dietary requirements in domestic animals, are not appropriate for zoo or endangered animals [13]. Improved approaches are needed to investigate biological species conservation on a large scale [14,15]. Fecal metabolomics, as a non-invasive and insightful method, is a promising new candidate to assist with the evaluation of the digestive processes within wildlife. The world’s tallest ruminant, the giraffe (*Giraffa camelopardalis*), is currently listed as a “vulnerable” species on the International Union for Conservation of Nature (IUCN) Red List [16]. Its unique anatomy is also reflected in its intricate ruminant digestive properties. Being selective browsers, this species has evolved unique ways to effectively maximize the absorption of dietary nutrients and thereby maintain the critical energy balance needed for the upkeep of their metabolism. Fecal metabolomics could provide insight into the unique chemical fingerprints that specific cellular processes leave behind [9] within this vulnerable species. Especially in environments that are still novel in its use, untargeted metabolomic studies are key in discovering potential metabolome and biochemical changes underlying the response to internal and/or external stimuli [9]. Combined with the assessment of environmental matrices, metabolomics can be used as an effective tool for the assessment of animal welfare, diet, demography and behavior and for the management of protected areas globally [17].

By making use of non-invasive fecal collection, the current study investigated the fecal metabolome present in giraffes at the end of the ruminant browser digestive processes. The only other known study investigating the metabolome of giraffe excretion products was conducted on urine that was collected from giraffes housed in captivity [18]. By using untargeted metabolomics through the application of comprehensive gas chromatography time-of-flight mass spectrometry (GCxGC/TOF-MS), this study primarily aims to provide the first-ever insights on the impact that external stimuli, such as supplemental feeding, seasonal variation and sex, have on the fecal metabolome composition of healthy, free-roaming giraffes.

## 2. Materials and Methods

### 2.1. Sample Collection

From July 2021 to June 2023, more than 300 fresh giraffe fecal samples were collected from six different locations and thirteen different giraffes in the central Free State, South Africa. To determine the differences between the metabolomes related to seasons and dietary supplements, 26 fecal samples (n = 26) were selected. All the populations were free-roaming and occurred within a 60 km radius from the city of Bloemfontein, which is located within the central Free State Province (South Africa). The location’s vegetation types include Bloemfontein Dry Grassland (Gh 5), Western Free State Clay Grassland (Gh 9) and Vaal-Vet Sandy Grassland (Gh 10) [19]. Supplemental feeding (SF) and natural available vegetation (NAV) feeding practices were followed at the different locations, which are listed in Table 1. Fecal collections occurred from 2021 to 2023 during the winter, dry (June–August), spring, dry (September–October), summer, wet (November–February) and autumn, wet (March–May) months. Metabolic analysis was also performed for the grouping of different seasons, such as dry (June to October) and wet (November to May), as listed in Table 1.

Fecal samples were handled similarly from collection to analysis. The collections were carried out directly after defecation to account for environmental factors that may affect the chemical and biological constituents of the feces. Fresh, wet samples were collected from the ground with a gloved hand and placed within plastic slider storage bags [20] and kept in a fridge (between 2 °C and 4 °C) for a maximum of three days, whereafter they were vacuum sealed and stored below freezing point until transportation to the North-West University (NWU), Centre for Human Metabolomics, South Africa, for metabolomics analysis. Immediate freezing of the fecal samples upon collection would be the best practice for preservation until analysis [21]. The lag time between collection and freezing is seen as one of the limitations of the current study but for logistical reasons, this condition was not feasible in the field [21].

Samples were given codes and were randomly analyzed in two batches. As with most free-roaming ungulate studies, our study design was unbalanced [22]. The results (n = 26) were pooled to differentiate between the habitats in which the management provides (SF) and does not provide supplemental feed (NAV), respectively, as listed in Table 1. The supplemental feed (SF) mainly comprised manufactured game pellets containing, in general, crude protein (minimum 100 g/kg), estimated energy (minimum 8 MJ/kg), moisture (maximum 120 g/kg), fat (minimum 30 g/kg), fiber (maximum 250 g/kg), calcium (maximum 10 g/kg) and phosphorous (minimum 4 g/kg) [23,24]. The provision of supplemental feed at Locations 1, 4 and 5 was conducted all year round. The samples were also pooled per month to compare the differences in the fecal metabolome during the dry (June to October) and wet (November to May) seasons (n = 9), as listed in Table 1.

### 2.2. Sample Preparation and Extraction

For sample preparation, a gas chromatography/time-of-flight mass spectrometry (GC/TOFMS) method used previously was applied, with slight variations [25]. The fecal sample (approximately 100 mg) was weighed off in a 5 mL Safelock microcentrifuge tube. The samples were ultrasonicated with 800 uL of methanol and 200 uL of 3-phenylbutyric acid (100ppm) in water (as internal standard) for 30 min and vortex mixed for 30 s. The sample mixtures were then centrifuged at 18,000 × g for 15 min at room temperature. The supernatant was transferred to a 2 mL glass vial and dried at 50 °C under a gentle stream of nitrogen gas. Toluene (100 uL) was added to the dry residue and evaporated at 50 °C under nitrogen gas to remove traces of water. The dried metabolic extract was then oximated with 50 μL of methoxyamine hydrochloride in pyridine (20 mg/mL) (Merck, Darmstadt, Germany) at 50 °C for 2 h. Following oximation, 100 uL of N, O-Bis (trimethylsilyl) trifluoroacetamide (BSTFA) with 1% trimethyl chlorosilane (TMCS) (Sigma-Aldrich, St. Louis, MO, USA) was added, and the mixture was incubated at 50 °C for 45 min to form trimethylsilyl (TMS) derivatives. The derivatized sample extracts were transferred to 2 mL glass vials containing inserts. For quality control purposes, a fatty acid methyl ester standard mixture, system blank (no injection prior to the analytical run) and extraction blank (following the entire sample preparation procedure but using a vial containing only methanol and no sample matrix), respectively, were injected four times during each batch [26]. All organic solvents used were ultra-pure Burdick & Jackson brands (Honeywell International Inc., Muskegon, MI, USA).

### 2.3. Comprehensive Gas Chromatography Time-of-Flight Mass Spectrometry (GCxGC/TOFMS Analysis)

One microliter of each sample extract was injected (1:5 split ratio) into a Pegasus 4D GCxGC-TOFMS machine (Leco Corporation, St. Joseph, MI, USA), utilizing an Agilent 7890A gas chromatographer (Agilent, Atlanta, GA, USA) coupled to a time-of-flight mass spectrometer (TOFMS) (Leco Corporation, St. Joseph, MI, USA) equipped with a Gerstel Multipurpose Sampler (Gerstel GmbH & co. KG, Eberhard-Gerstel- Platz 1, D-45,473 Mülheim an der Ruhr).

First, dimensional separation was achieved with an Rxi-5Sil-MS primary column (28.2 m, 0.25 mm internal diameter and 0.25 μm film thickness) (Restch GmbH & Co. KG, Haan, Germany), and an Rxi-17 capillary column (1.320 m, 0.25 mm internal diameter and 0.25 μm film thickness) was fitted as the secondary column (Restch GmbH & co. KG, Haan, Germany). The front inlet temperature was held at a constant 270 °C for the entire run, ensuring rapid vaporization. For the primary oven, an initial GC oven temperature was set at 70 °C for 2 min followed by an increase in the oven temperature of 4 °C/min to a final temperature of 300 °C, which was held for 2 min. The secondary column oven temperature was set at 85 °C for 2 min and then increased by 4.5 °C/min until it reached a final temperature of 310 °C, at which it was maintained for an additional 4.5 min. The initial temperature of the modulator was 100 °C for 2 min, followed by a 4 °C/min increase to a final temperature of 310 °C that was held for 12 min. To control the effluent from the primary onto the secondary column, cryo-modulation and a hot pulse of nitrogen gas of 0.5 s every 3 s was used.

The acquisition delay for each run was 350 s, and the transfer line temperature was held at a constant 270 °C, with the ion source temperature at a constant 200 °C. The detector voltage was adjusted to 150 V and offset with a filament bias of −70 eV. Spectra were collected in scan mode from 50 to 950 m/z at an acquisition rate of 200 spectra per second. Mass spectral deconvolution, peak alignment and peak identification were performed using Leco Corporation’s ChromaTOF software (version 4.71). Mass spectral deconvolution was performed at a signal-to-noise ratio of 350, with a minimum of three apexing peaks. To eliminate the effect of retention time shifts and to create a data matrix containing the relative abundance of all compounds present in all samples, peaks with similar mass spectra and retention times were aligned using Statistical Compare, a package of ChromaTOF. Mass fragmentation patterns and their respective retention times were screened against commercially available National Institute of Standards and Technology (NIST) spectral libraries (mainlib, replib) for peak annotation, with a similarity setting of at least 80% [27].

### 2.4. Data Analysis

The raw GCxGC-MS data, including peak areas and annotations for each sample, were exported to MS Excel Version 2409 [28]. Compounds with the same name and unique mass were consolidated (summed). Contaminant compounds were identified by comparing the compounds detected in the extraction blank and system blank to those detected in the samples. Peaks detected in the system blanks were deleted from the dataset if their average concentration in the samples were 20 times or less than the average in the system blanks. Compounds present at concentrations 20 times or higher in the average of the samples, compared to the system blanks, were kept in the dataset and marked for future reference. Peaks detected in the extraction blanks were deleted from the dataset if the mean concentration in the samples was three times or less than that in the blanks [26]. If a compound was detected in both the blanks and samples but with an average concentration that was three times or more in the samples compared to the blanks, it was retained in the dataset and marked for future reference. The samples were quantified relative to the internal standard.

Statistical analysis was conducted using MetaboAnalyst (version 6.0) [29], a web server for metabolomics data analysis and interpretation based on the statistical program “R” Version 4.4.1 [30]. The data were normalized to the sample median, log transformed and auto-scaled prior to processing. To evaluate the cofounding effect of the available biological information, the processed data were analyzed by assigning the samples to different subgroups, including SF versus NAV, samples collected during the wet season versus the dry season and male versus female. Principal component analysis (PCA) was used to summarize the data based on the various subgroup comparisons: (a) giraffes provided with SF and only NAV, (b) wet and dry seasons for a single adult male giraffe, (c) fecal samples collected from six male and seven female giraffes from all locations, (d) fecal samples collected from five male and seven female giraffe from different feeding practices and (e) fecal samples collected from one adult male (I) and the sub-adult male (II) at Location 1. PCA is an unsupervised method that aims to find the directions that best explain the variance in a dataset (X) without referring to class labels (Y). The data are summarized into much fewer variables called scores, which are weighted average of the original variable. These scores can then be plotted in order to give a visual over-view of the samples and how they correlate with each other [11]. For univariate analyses, analysis of variance (ANOVA), including Student’s t-test and Fisher’s least significant difference (LSD), and fold-change (FC) analyses were performed [29]. Metabolites were identified as statistically significant differential compounds for the said group comparisons if they had a *p* < 0.05 and Log^2^ (FC) of > 4. Heatmaps (Pearson distance calculation and ward clustering algorithm) of the significant compounds were also created.

## 3. Results

### 3.1. Population

A total of 26 fecal samples from 13 different free-roaming giraffes were included in this study. The principal composition of the subgroups for analysis (feeding practice, season and sex) is described in Table 1.

The sample size was very small, and therefore, it should be noted that this is a pilot study to determine if metabolomics can be used as a tool to investigate the effects of age, sex, feeding practice and seasonal variation on the giraffe fecal composition.

### 3.2. Overview of the Metabolomic Data and Subgroup Comparisons

**Figure 1:**
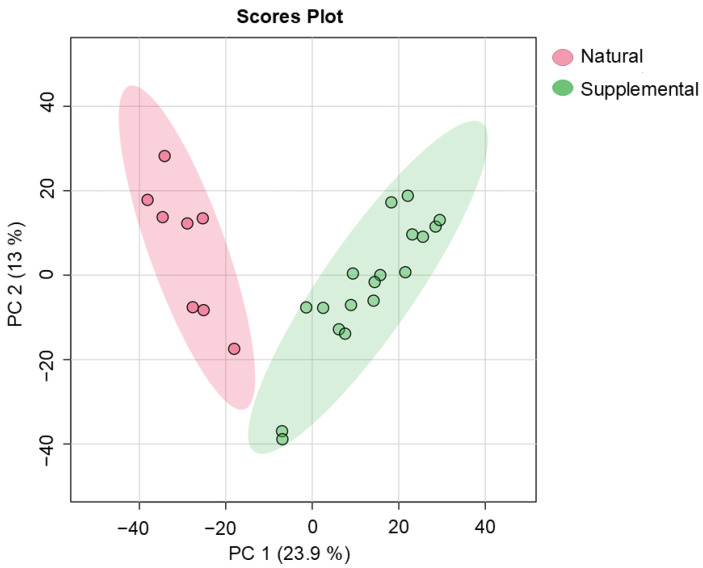
Principal component analysis scores plot (PC 1 and 2, with the variation explained in brackets) of fecal metabolite profiles of giraffes provided with supplemental feed (SF) and giraffes only feeding on the natural available vegetation (NAV). PC: principal component.

**Figure 2:**
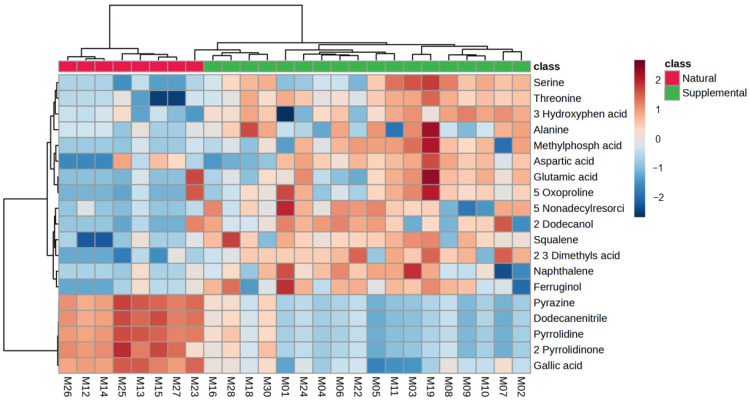
Heatmap (Pearson distance calculation and ward clustering algorithm) of the significant (*p* < 0.05) annotated metabolites with a fold-change test (Log^2^ [FC]) of >4, when comparing data from giraffes receiving supplemental feed (SF) and giraffes only feeding on the natural available vegetation (NAV).

Clear variations between the data obtained from the SF and NAV giraffe samples were seen on the PCA scores plot, which is shown in Figure 1. Significantly higher concentrations of a number of compound classes (such as amino acid-related and carbohydrate-related metabolites), which are summarized in Table 2 and Figure 2, were noted for giraffes receiving SF compared to the NAV subgroup.

**Figure 3:**
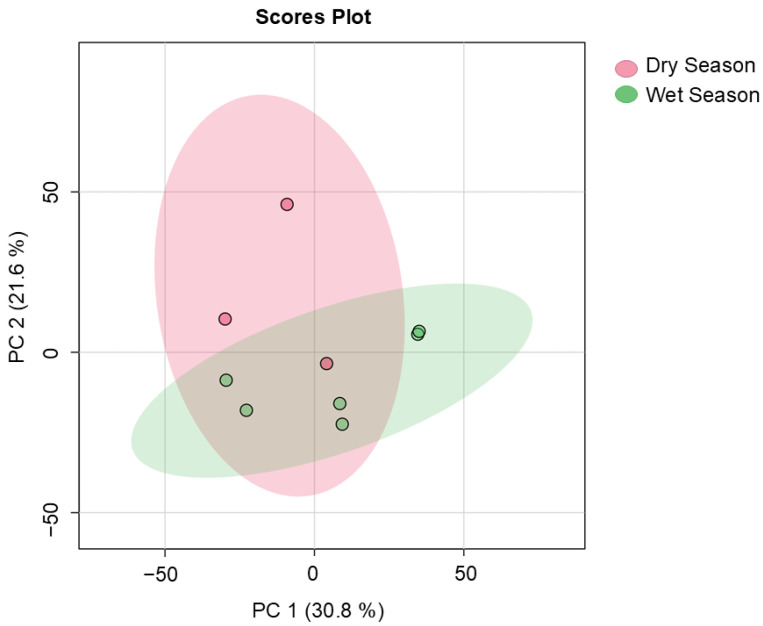
Principal component analysis scores plot (PC 1 and 2, with the variation explained in brackets) of fecal metabolite profiles of a single giraffe during the wet and dry seasons over a two-year period. PC: principal component.

**Figure 4:**
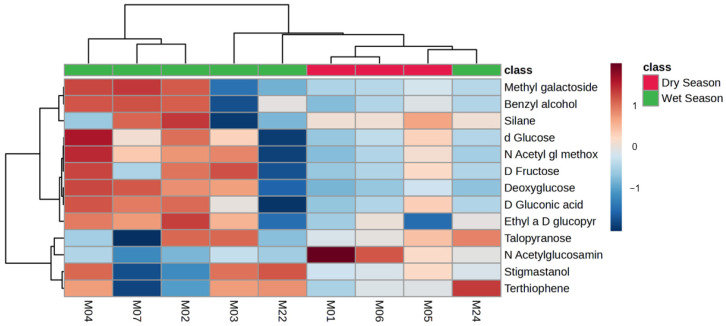
Heatmap (Pearson distance calculation and ward clustering algorithm) of the annotated metabolites with a fold-change test (Log^2^ [FC]) of >4 when comparing seasonal variations (wet and dry seasons) from feces collected from a single adult male giraffe at a central Free State location over a two-year period.

A clear separation between the data obtained from the wet and dry seasons, as identified from a single adult male giraffe (indicated as “I” in Table 1) was seen, as shown in Figure 3. Higher concentrations of a number of compound classes (carbohydrate-related metabolites and organic compounds) during the wet season were noted in Table 3. A heatmap of the compounds with an FC > 4 (wet versus dry comparison) identified from giraffe feces is visually summarized in Figure 4.

**Figure 5:**
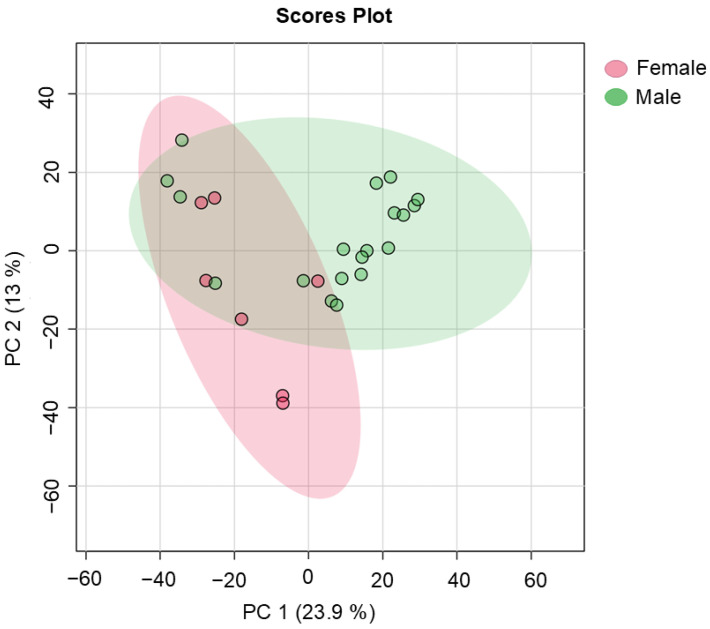
Principal component analysis scores plot (PC 1 and 2, with the variation explained in brackets) of fecal metabolite profiles identified for all male and female giraffes in free-roaming populations at six different locations in the central Free State over a two-year period. PC: principal component.

**Figure 6:**
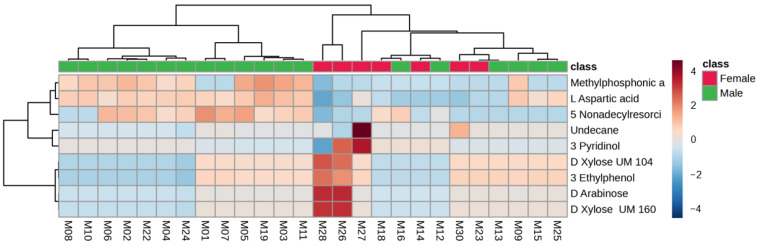
Heatmap (Pearson distance calculation and ward clustering algorithm) of the significant (*p* < 0.05) annotated metabolites with a fold-change test (Log^2^ [FC]) of >4 when comparing male and female giraffes’ metabolite profiles.

Variations in profiles were also identified for the metabolites from fecal samples collected from the six males and seven females, as shown in Figure 5. Differences, such as higher levels of carbohydrate-related metabolites, alkanes and phenols for female giraffes (compared to males) are noted in Table 4 and Figure 6.

**Figure 7:**
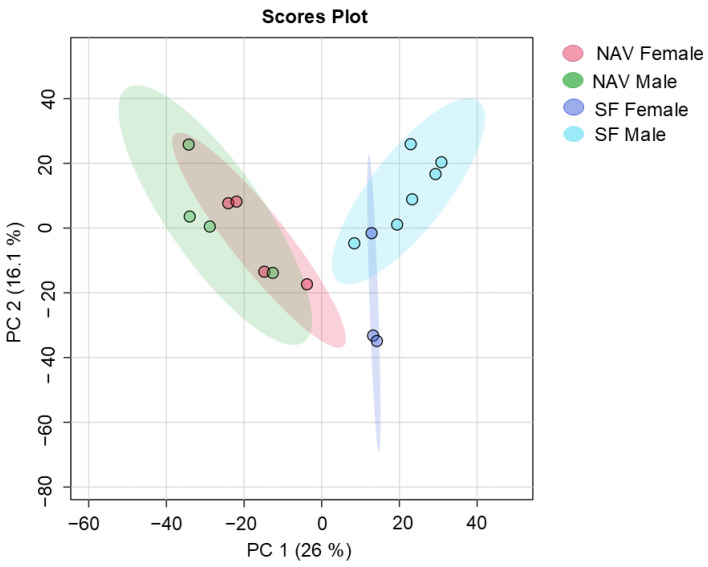
Principal component analysis scores plot (PC 1 and 2, with the variation explained in brackets) of fecal metabolite profiles identified for male and female giraffes provided with supplemental feed (SF) and giraffes only feeding on the natural available vegetation (NAV) at different locations in the central Free State over a two-year period. Metabolomic data from the adult male giraffe at Location 1 were excluded. PC: principal component.

**Figure 8:**
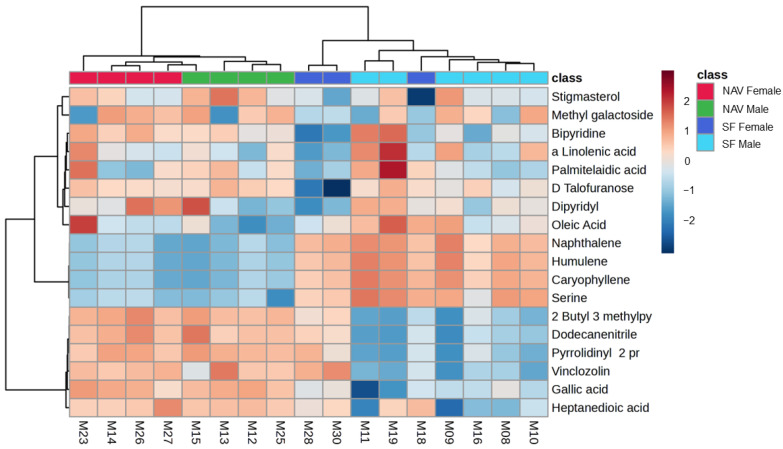
Heatmap (Pearson distance calculation and ward clustering algorithm) of the significant annotated metabolites with an f-value > 13 (ANOVA; Fisher’s LSD) when comparing male and female giraffes provided with supplemental feed (SF) and giraffes only feeding on the natural available vegetation (NAV) at different locations in the central Free State over a two-year period. Metabolomic data from the adult male giraffe at Location 1 were excluded.

Clear separate metabolite profiles were also identified for male and female giraffes provided with SF or NAV at different locations in the central Free State over a two-year period, as shown in Figure 7. Metabolomic data from the adult male giraffe (“I”) at Location 1 were excluded for this analysis in an attempt to balance the male and female representation. A heatmap of the of the significant annotated metabolites with an f-value > 13 (ANOVA; Fisher’s LSD) when comparing the male and female subgroups is visually summarized in Figure 8 and Table 5.

**Figure 9:**
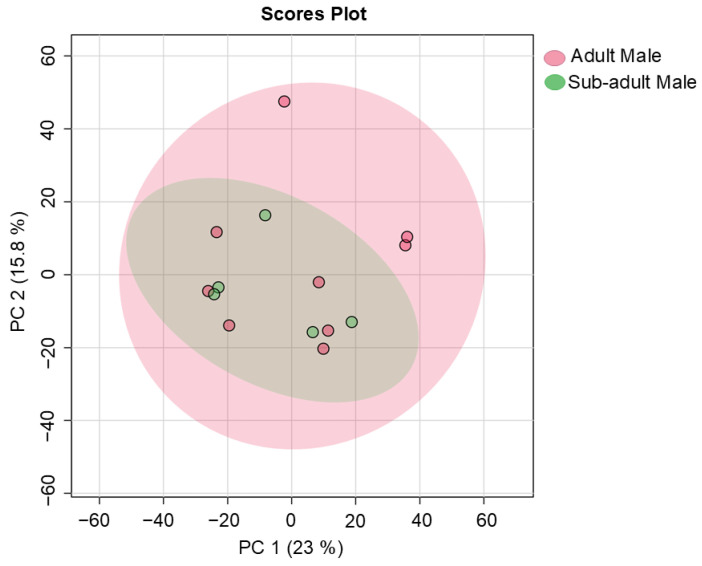
Principal component analysis scores plot (PC 1 and 2, with the variation explained in brackets) of fecal metabolite profiles identified for an adult male giraffe and sub-adult male giraffe at the same location in the central Free State over a two-year period. PC: principal component.

**Figure 10:**
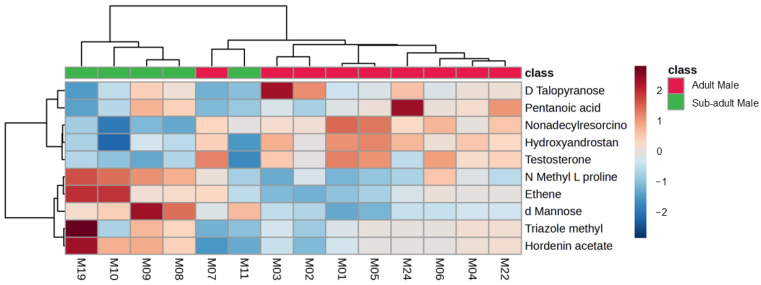
Heatmap (Pearson distance calculation and ward clustering algorithm) of the annotated metabolites with a fold-change test (Log^2^ [FC]) of >3), when comparing the metabolite profiles of an adult male giraffe and sub-adult male giraffe at the same location.

To investigate possible differences between adult and sub-adult individuals, a comparison was performed between the fecal samples collected from the one adult male (I) and the sub-adult male (II) at Location 1 (Table 1). A definite overlap in the data obtained from the adult male giraffe and the sub-adult was seen, as shown in Figure 9. Higher concentrations in male reproduction hormone metabolites, such as 3-hydroxyandrostan-17-one (androsterone) and testosterone, were noted for the adult male giraffe, as shown in Table 6. A heatmap of the compounds with an FC > 3 (adult male versus sub-adult male comparison) identified from giraffe feces is visually summarized in Figure 10.

The identified differentiating metabolites are hypothesis generating and can be used to guide the choice of future, more targeted analyses on giraffe health quantification. The following box–whisker plots were created and form part of the Supplementary Materials: Figure S1: Significant (*p* < 0.05) metabolites with an FC test (Log^2^ [FC] > 4) identified for giraffe receiving SF or only NAV; Figure S2: Significant (*p* < 0.05) metabolites with an FC test (Log^2^ [FC] > 4) identified for the wet and dry seasons from feces collected from a single adult male giraffe; Figure S3: Significant (*p* < 0.05) metabolites with an FC test (Log^2^ [FC] > 4) identified for male and female giraffes.

## 4. Discussion

The natural browsing diet of free-roaming giraffes includes material from trees and shrubs, such as leaves, shoots, flowers, fruits and twigs [31,32,33,34,35,36,37], and is rich in crude protein and lignin contents [38,39,40]. These materials are ingested, and in the rumen, the foregut fermentation process starts. Similar to other ruminants, giraffes depend on the symbiotic relationship with microorganisms in the rumen and the rest of the digestive system to digest and absorb energy such as volatile fatty acids (VFAs), which are locked in cellulose and hemicellulose [39,41,42,43,44,45,46,47], to nurture the animal, along with a great variety of excreted fecal metabolites as an end product [42,48,49,50]. As a result, feces are a mixture of undigested material, microbiota, enzymes and metabolites [51]. It is estimated that 40% of the daily energy is derived from VFAs and produced by microbial fermentation in the rumen [52] and that 60% is from the intestinal digestion of glucose [36].

Although several studies have been conducted on ruminant fecal metabolomes [6,53], most of the research focused on grazers (especially domestic livestock in animal production studies) and not browsers. Some fundamental differences exist between foraging and browsing materials, which are consumed by grazers and browsers, respectively, with regard to cell structure, plant chemistry and animal metabolism [54,55,56]. As a result, no direct relationships between the metabolomics of grazers and browsers can be drawn. Basic microbial fermentation and the created by-products do, however, remain the same for different types of ruminants. These would include short-chain fatty acids, aldehydes, alcohols, phenols, ketones, esters, carbohydrate-related metabolites and amino, propionic and butyric acids [57], only differing in concentrations and profiles based on the available diet and animal phenotype [58].

During the current study, clear variations in the metabolome profiles were noted for giraffe receiving SF and those only browsing on NAV, as illustrated in Figure 1. Comparatively, significantly higher concentrations of amino acid-related metabolites (threonine, oxoproline, serine, aspartic acid, alanine, glutamic acid and hydroxyphenylacetic acid) were identified for giraffes receiving SF, as shown in Table 2 and Figure 2. Amino acid-related metabolites are commonly used in the biosynthesis of proteins [29,59] and, in this case, can be indicative of the increased consumption of additional dietary supplements (SF) available from the commercially manufactured game pellets, which contain a mixture of forage, grain and roughage products, sugar by-products, proteins, minerals and trace mineral vitamins [23,24]. Significantly higher concentrations of organic compounds (pyrrolidine, pyrazine, gallic acid and pyrrolidinone) were, however, noted for giraffes with a diet of only NAV, as shown in Table 2 and Figure 2. Most likely, the greater availability and variety in natural vegetation options at Locations 2, 3 and 6 can be attributed to these significantly higher concentrations.

A significantly higher concentration in methylphosphonic acid (MPA) in the feces collected from SF giraffes (compared to NAV) was also noted. MPA is a breakdown product of organophosphorus compounds, which are found in some pesticides [60]. Its presence in feces could be due to environmental exposure or dietary sources [61]. Its increased detection as an end product from the digestive system of giraffes provided with SF raises questions about the safety of the SF or even the giraffes’ exposure to commercial farming practices at Locations 1, 4 and 5 (Table 1).

Fecal metabolome variations were identified in samples collected from a single adult male giraffe (Location 1, SF) during the wet and dry seasons, as shown in Figure 3. Differences, such as comparatively higher carbohydrate-related metabolites and organic compounds during the wet season were noted in Table 3 and Figure 4. This increase could be generally related to an increase in the available dietary energy during the spring and summer (wet) seasons [29,32,59], such as the growth of new plant shoots and flowers [36,62]. An increase in the sample size would most definitely aid in improving the statistical significance of a seasonal metabolome profile change and is noted as one of the limitations of the current study. A similar higher value for terthiophene, a pesticide [60], was noted from the feces of the adult male giraffe towards the end of the wet season. Its presence in feces could be due to environmental exposure or the dietary source [61]. Its increased detection as a digestion end product at the end of the wet season (M24, March 2023, Table 1) supports a possible increased environmental exposure or build-up coinciding with the continuous use of pesticides during the wet season, originating from surrounding agricultural practices.

Distinct variations in profiles were also identified for the metabolites from fecal samples collected from the six males and seven females, as shown in Figure 5. Differences, such as higher levels of carbohydrate-related metabolites, undecane and 3-ethylphenol for female giraffes (compared to males) were noted in Table 4 and Figure 6. Due to their ability to seek out the most nutritious plant parts, giraffes are known to be nonseasonal breeders [63]. Although the gestation time for the giraffe females were not determined for this study, it could be that the significantly higher concentrations of carbohydrate-related metabolites (xylose and arabinose) are indicative of an increased metabolism coinciding with the gestation period or milk production for lactation. Also of interest is the higher level of undecane. This organic compound (an alkane) has been found to be a mild sex attractant for various types of insects, such as moths and cockroaches [64], and an alert signal for a variety of ants [60,65]. During this study, undecane was seen to have the highest concentration in females with a lower concentration of xylose and arabinose metabolites in their feces. This observation leads to hypothesizing that undecane in the presence of female giraffe feces could be indicative of reproductive status, as previously found with fecal steroid analysis [63,66].

Excluding the metabolomic data from the adult male giraffe at Location 1 and comparing the profiles of the remaining five males and seven females still indicated clear separate metabolite profiles at the different locations, as summarized in Figure 7 and Figure 8 and Table 5. The concentration of Vinclozolin, again, a pesticide, was higher in the female (SF and NAV) subgroups when compared to the male subgroups. This compound has a antiandrogenic activity that has proven to result in delays in mammalian meiocyte differentiation and follicle growth [67]

To investigate possible differences between adult and sub-adult individuals, a comparison was conducted between the fecal samples collected from the single adult male (I) and the sub-adult male (II) at Location 1 (Table 1). A definite overlap in the data obtained from the adult male giraffe and the sub-adult from the same location was seen, as shown in Figure 9 and Figure 10. A notable difference was the higher concentrations in male reproduction hormone metabolites, such as 3-hydroxyandrostan-17-one (androsterone) and testosterone [60], as noted for the adult male giraffe in Table 6. The measurement of testosterone metabolites in male vertebrate feces has been previously described [68], with higher levels occurring during the breeding season and in sexually mature males [69], which would also explain the findings of the current study.

## 5. Conclusions

This is the first study of its kind to investigate the fecal metabolome of free-roaming giraffes, as well as the effects that external factors, such as environmental exposures, feeding practices, seasonal variations, age and sex, have on it. The variations in the fecal metabolome profiles that were identified for all subgroups using untargeted metabolomics analysis assist in the development of future improved study designs to further clarify its significance along with associated metabolic pathways. This novel use of fecal metabolomics adds to the non-invasive toolbox of analyses that do not require capture, restraint, blood collection and consequent influential stress factors to free-roaming giraffes. Such advances are beneficial towards the conservation of wildlife species on a larger scale.

## Figures and Tables

**Table 1:** Overview of the samples collected and analyzed for the fecal metabolomes of 13 different giraffes provided with supplemental feeding (SF) and those only fed with the natural available vegetation (NAV) at different locations within the central Free State over a two-year period. The samples collected and analyzed for the fecal metabolome from a single adult male giraffe at a Location 1, across the wet and dry seasons over the two-year period, are also indicated.

Sample Code	Feeding Practice	Location	Age Class, Sex and Individual Number	Date(s) of Fecal Collection ^1^	Assigned Season ^2^
M01	Supplemental feeding	1	Adult male (I)	03, 18, 31 August 2021	Winter, dry
M03		1	Adult male (I)	09, 18, 28 March 2022	Autumn, wet
M05		1	Adult male (I)	01, 14, 22 June 2022	Winter, dry
M07		1	Adult male (I)	04, 23 November 2022	Summer, wet
M02		1	Adult male (I)	13 February 2022	Summer, wet
M04		1	Adult male (I)	05 and 27 April 2022	Autumn, wet
M06		1	Adult male (I)	11, 24, 25 August 2022	Winter, dry
M22		1	Adult male (I)	07 April 2023	Autumn, wet
M24		1	Adult male (I)	20 March 2023	Autumn, wet
M09		1	Sub-adult male (II)	11 February 2022	N/A ^2^
M11		1	Sub-adult male (II)	13, 19, 27 April 2022	N/A
M19		1	Sub-adult male (II)	07 April 2023	N/A
M08		1	Sub-adult male (II)	04, 09, 23 August 2021	N/A
M10		1	Sub-adult male (II)	09, 18 March 2022	N/A
M16		4	Adult male (III)	10 March 2023	N/A
M18		4	Adult female (IV)	10 March 2023	N/A
M28		5	Adult female (V)	13 September 2022	N/A
M30		5	Adult female (V)	09 October 2022	N/A
M15	Natural available vegetation	2	Adult male (VI)	10 March 2023	N/A
M23		2	Adult female (VII)	22 April 2023	N/A
M14		2	Adult female (VIII)	10 March 2023	N/A
M25		3	Adult male (IX)	10 April 2023	N/A
M27		3	Adult female (X)	10 April 2023	N/A
M26		3	Adult female (XI)	10 April 2023	N/A
M13		6	Adult male (XII)	16 February 2023	N/A
M12		6	Adult male (XIII)	16 February 2023	N/A

^1^ If collected on more than one date, the samples were pooled per month. ^2^ Wet versus dry season comparison was only performed for a single adult male giraffe (indicated as “I”).

**Table 2:** Statistically significant (*p* < 0.05) fecal metabolites identified when comparing giraffes provided with SF and giraffes only feeding on the NAV during a two-year period.

Class	Significant Differential Compounds (SF and NAV)	FC	Log^2^ (FC)	Higher (↑) or Lower (↓) in SF	*p*-Value
Amino acid related	Threonine	42.55	5.41	↑	*p* < 0.05
Amino acid related	5-Oxoproline	18.67	4.22	↑	*p* < 0.05
Amino acid related	Serine	18.49	4.21	↑	*p* < 0.05
Amino acid related	Aspartic acid	17.68	4.14	↑	0.003
Amino acid related	Alanine	17.39	4.12	↑	0.0011
Amino acid related	Glutamic acid	17.38	4.12	↑	0.0002
Amino acid related	3-Hydroxyphenylacetic acid	17.32	4.11	↑	0.002
Organic compound	Pyrrolidine	0.029	−5.10	↓	*p* < 0.05
Organic compound	Pyrazine	0.03	−4.91	↓	*p* < 0.05
Organic compound	Squalene	31.36	4.97	↑	*p* < 0.05
Organic compound	Gallic acid	0.05	−4.30	↓	*p* < 0.05
Organic compound	Naphthalene	17.49	4.13	↑	0.0002
Organic compound	2-Pyrrolidinone	0.058	−4.31	↓	*p* < 0.05
Dicarboxylic acid	2,3-Dimethylsuccinic acid	22.76	4.51	↑	*p* < 0.05
Fatty alcohol	2-Dodecanol	44.95	5.49	↑	0.0002
Fatty nitriles	Dodecanenitrile	0.035	−4.83	↓	*p* < 0.05
Phenol	Ferruginol	35.69	5.16	↑	*p* < 0.05
Phenol	5-Nonadecylresorcinol	71.85	6.17	↑	0.0003
Inorganic compound	Methylphosphonic acid	77.35	6.27	↑	0.0004

FC: fold change; NAV: natural available vegetation; SF: supplemental feeding.

**Table 3:** Significant differential fecal metabolites (with regards to fold change) identified from samples collected from a single adult male giraffe during the dry and wet seasons over a two-year period. No compounds were classified as significant with the *t*-test analyses (*p* < 0.05).

Class	Significant Differential Compounds (Wet and Dry Seasons)	FC	Log^2^ (FC)	Higher (↑) or Lower (↓) in the Wet Season
Amino acid related	N-Acetylglucosamine	18.43	4.20	↓
Carbohydrate related	Deoxyglucose	0.02	−5.87	↑
Carbohydrate related	D-Fructose	0.04	−4.51	↑
Carbohydrate related	N-Acetyl glucosamine methoxime	0.05	−4.43	↑
Carbohydrate related	Talopyranose	0.05	−4.25	↑
Carbohydrate related	d-Glucose	0.06	−4.09	↑
Carbohydrate related	Methyl galactoside	0.001	−9.08	↑
Carbohydrate related	D-Gluconic acid	0.06	−4.12	↑
Carbohydrate related	Ethyl à-D-glucopyranoside	0.06	−4.01	↑
Organic compound	Benzyl alcohol	0.03	−5.18	↑
Organic compound	Terthiophene	0.03	−4.92	↑
Sterol	Stigmastanol	0.03	−4.98	↑
Inorganic compound	Silane	0.06	−4.19	↑

FC: fold change.

**Table 4:** Statistically significant fecal metabolites identified from samples from male and female giraffes’ feces, collected from July 2021 to June 2023 from different locations in the central Free State.

Class	Significant Differential Compounds (Males and Females)	FC	Log^2^ (FC)	Higher (↑) or Lower (↓) in Females	*p*-Value
Amino acid related	L-Aspartic acid	0.03	−5.06	↓	0.0002
Carbohydrate related	D-Arabinose	15598	13.93	↑	0.001
Carbohydrate related	D-Xylose UM 160	1587.5	10.63	↑	0.001
Carbohydrate related	D-Xylose UM 104	99.56	6.637	↑	0.001
Organic compound	Pyrrolidine	21.61	4.434	↑	0.0003
Organic compound	Methylphosphonic acid	0.04	−4.477	↓	NS
Alkanes	Undecane	18.50	4.21	↑	0.0002
Phenol	3-Ethylphenol	50.45	5.66	↑	0.001
Phenol	5-Nonadecylresorcinol	0.055	−4.19	↓	0.001

FC: fold change; NS: not significant; UM: unique.

**Table 5:** Differential significant annotated metabolites with an f-value > 13 (ANOVA; Fisher’s LSD), when comparing male and female giraffes provided with supplemental feed (SF) and giraffes only feeding on the natural available vegetation (NAV) at different locations in the central Free State over a two-year period. Metabolomic data from the adult male giraffe at Location 1 were excluded.

Class	Significant Differential Compounds (SF Females, SF Males, NAV Females and NAV Males)	f-Value	*p*-Value	Higher (↑) or Lower (↓) in SF Females Compared to SF Males ^1^	Higher (↑) or Lower (↓) in NAV Females Compared to NAV Males
Amino acid related	Serine	14.105	<0.001	↓	↑
Carbohydrate related	D-Talofuranose	14.235	<0.001	↓	↓
Fatty nitriles	Dodecanenitrile	27.559	<0.001	↑	↑
Organic compound	Bipyridine	70.445	<0.001	↓	↑
Organic compound	2-Butyl-3-methylpyrazine	27.936	<0.001	↑	↑
Organic compound	Pyrrolidinyl-2 propanone	27.675	<0.001	↑	↓
Organic compound	Caryophyllene	23.22	<0.001	↓	↑
Organic compound	Vinclozolin	21.978	<0.001	↑	↑
Organic compound	Heptanedioic acid	20.719	<0.001	↑	↑
Organic compound	Humulene	18.458	<0.001	↓	↑
Organic compound	Stigmasterol	18.444	<0.001	↓	↓
Organic compound	Gallic acid	14.032	<0.001	↑	↑
Organic compound	Naphthalene	13.417	<0.001	↓	↑

^1^ ANOVA: analysis of variance; LSD: least significant difference; NAV: natural available vegetation; SF: supplemental feeding.

**Table 6:** Differential fecal metabolites (fold-change test (Log^2^ [FC]) of >3) identified from fecal samples collected from an adult male giraffe and sub-adult male from July 2021 to June 2023 at the same location in the central Free State.

Class	Significant Differential Compounds (Adult and Sub-Adult Males)	FC	Log^2^ (FC)	Higher (↑) or Lower (↓) in the Adult Male	*p*-Value
Amino acid related	L-proline	0.11	−3.14	↓	NS
Carbohydrate related	d-Mannose	0.086	−3.55	↓	NS
Carbohydrate related	D-Talopyranose	9.05	3.18	↑	NS
Organic compound	Triazole	0.039	−4.70	↓	NS
Organic compound	Nonadecylresorcinol	18.50	4.21	↑	NS
Organic compound	3-hydroxyandrostan-17-one (androsterone)	14.02	3.81	↑	<0.001
Organic compound	Testosterone	13.77	3.78	↑	NS
Organic compound	Ethene	0.10	−3.26	↓	NS
Organic compound	Pentanoic acid	10.87	3.44	↑	NS
Organic compound	Hordenin	0.11	−3.14	↓	NS

NS: not significant.

## Data Availability

Dataset available on request from the authors.

## Supplementary Materials

The following supporting information can be downloaded at: https://www.mdpi.com/article/10.3390/metabo14110586/s1. Figure S1: Significant (*p* < 0.05) metabolites with a fold-change test (Log^2^ [FC] > 4) identified for giraffe receiving SF or only NAV; Figure S2: Significant (*p* < 0.05) metabolites with a fold-change test (Log^2^ [FC] > 4) identified for the wet and dry seasons from feces collected from a single adult male giraffe; Figure S3: Significant (*p* < 0.05) metabolites with a fold-change test (Log^2^ [FC] > 4) identified for male and female giraffes. Other datasets can be made available by the authors on request.
