# Supplementary material for: First Insights into the Fecal Metabolome of Healthy, Free-Roaming Giraffes (Giraffa camelopardalis): An Untargeted GCxGC/TOF-MS Metabolomics Study
Source: Metabolites. 2024 Oct 28;14(11):586. doi: 10.3390/metabo14110586 (PMC11596133; doi:10.3390/metabo14110586)
Supplement: Supplementary file 1 [file metabolites-14-00586-s001.zip › metabolites-3226042-supplementary.pdf]

metabolites

MDPI

Article – Supplementary Material

First insights into the Faecal Metabolome of Healthy, Free-roaming Giraffe (*Giraffa camelopardalis*): an Untargeted GCxGC/TOF-MS Metabolomics study

Andri Grobbelaar [1], Gernot Osthoff [2], Ilse du Preez [3] and Francois Deacon [1,*]

[1] Department of Animal Sciences, Faculty of Natural and Agricultural Sciences. University of the Free State. PO Box 339, Bloemfontein, 9300, SA; andri.giraffe@gmail.com; deaconf@ufs.ac.za
[2] Department of Microbiology and Biochemistry, Faculty of Natural and Agricultural Sciences. University of the Free State. PO Box 339, Bloemfontein, 9300, SA; osthoffg@ufs.ac.za
[3] Centre for Human Metabolomics, North-West University, Potchefstroom, South Africa; ilse.dupreez@nwu.ac.za
* Correspondence: deaconf@ufs.ac.za; Tel.: (+27)514013554

Supplementary Material

The identified differentiating metabolites are hypothesis generating, and can be used to guide the choice of future, more targeted analysis on giraffe health quantification. The following Box-whisker plots will be made available online at http://www.mdpi.com/????, Figure S1: Significant ($p < 0.05$) metabolites with a fold change test (Log^2 [FC]> 4) identified for giraffe receiving SF or only NAV; Figure S2: Significant ($p < 0.05$) metabolites with a fold change test (Log^2 [FC]> 4) identified for the wet and dry seasons from faeces collected from one, adult male giraffe individual; Figure S3: Significant ($p < 0.05$) metabolites with a fold change test (Log^2 [FC]> 4) identified for male and female giraffes. Other data sets can be made available by the authors on request.

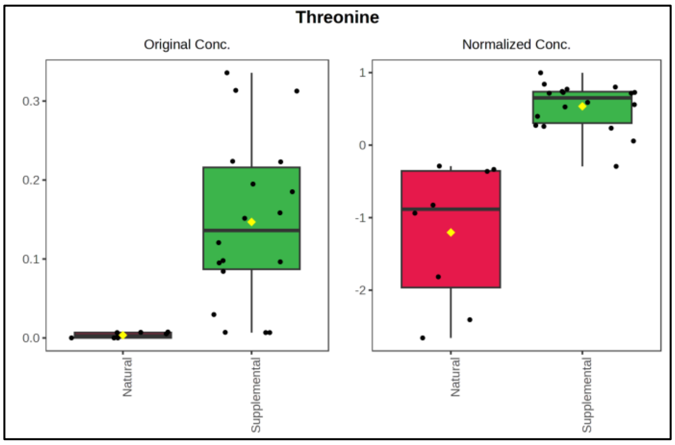
Threonine
Original Conc.
Normalized Conc.
0.3
0.2
0.1
0.0
1
0
-1
-2
Natural
Supplemental
Natural
Supplemental

(a)

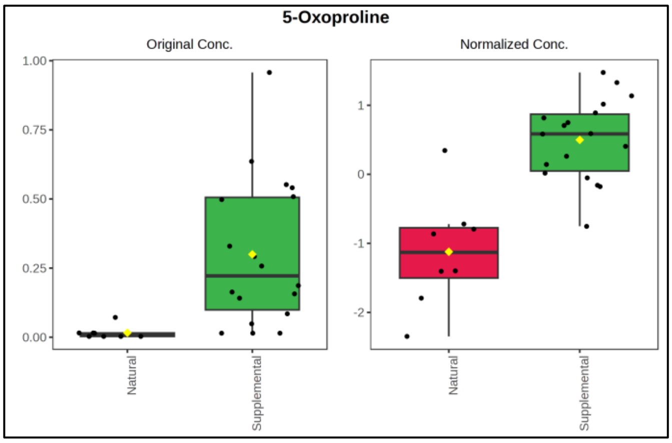
5-Oxoproline
Original Conc.
Normalized Conc.
1.00
0.75
0.50
0.25
0.00
1
0
-1
-2
Natural
Supplemental
Natural
Supplemental

(b)

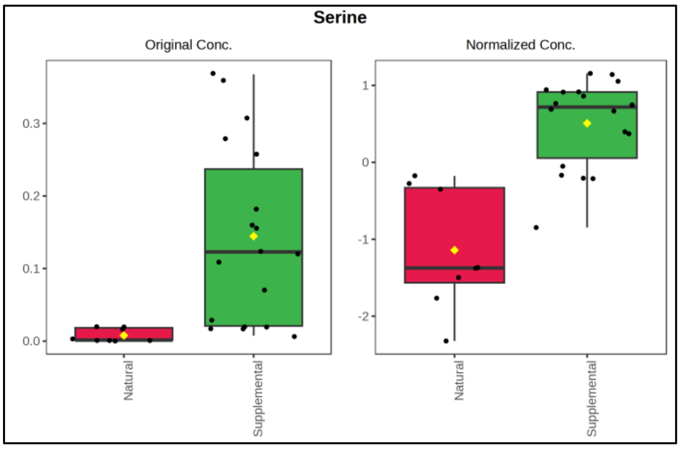
Serine
Original Conc.
Normalized Conc.
0.3
0.2
0.1
0.0
1
0
-1
-2
Natural
Supplemental
Natural
Supplemental

(c)

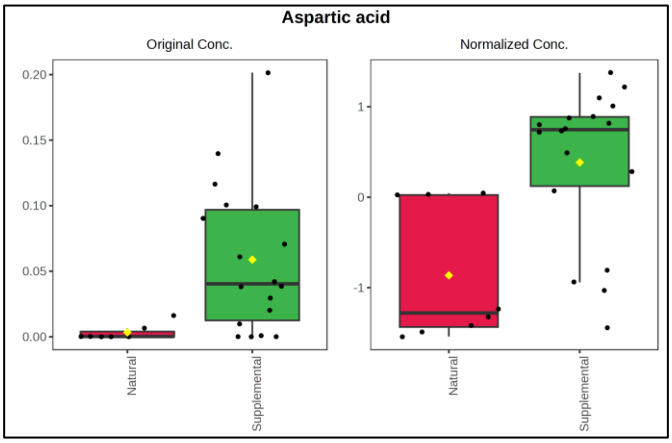
Aspartic acid
Original Conc.
Normalized Conc.
0.20
0.15
0.10
0.05
0.00
1
0
-1
Natural
Supplemental
Natural
Supplemental

(d)

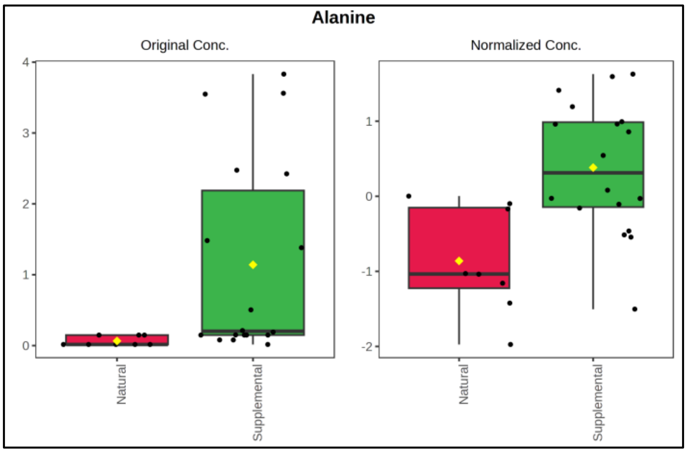
Alanine
Original Conc.
Normalized Conc.
4
3
2
1
0
1
0
-1
-2
Natural
Supplemental
Natural
Supplemental

(e)

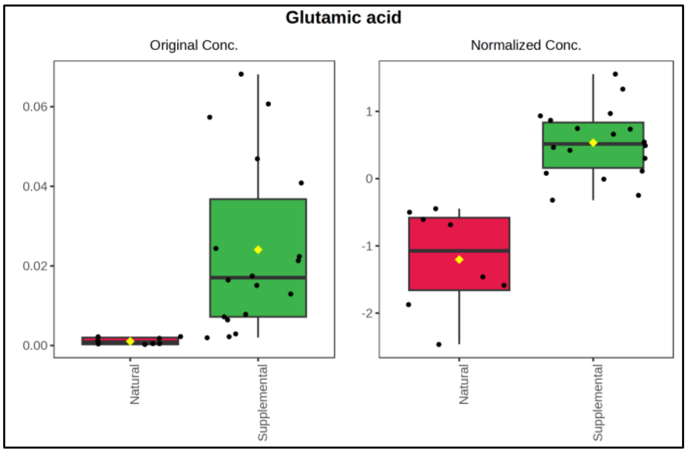
Glutamic acid
Original Conc.
Normalized Conc.
0.06
0.04
0.02
0.00
1
0
-1
-2
Natural
Supplemental
Natural
Supplemental

(f)

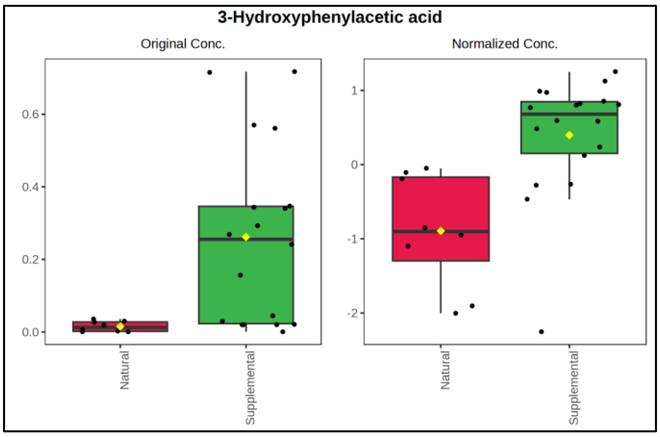
3-Hydroxyphenylacetic acid
Original Conc.
Normalized Conc.
Natural
Supplemental

(g)

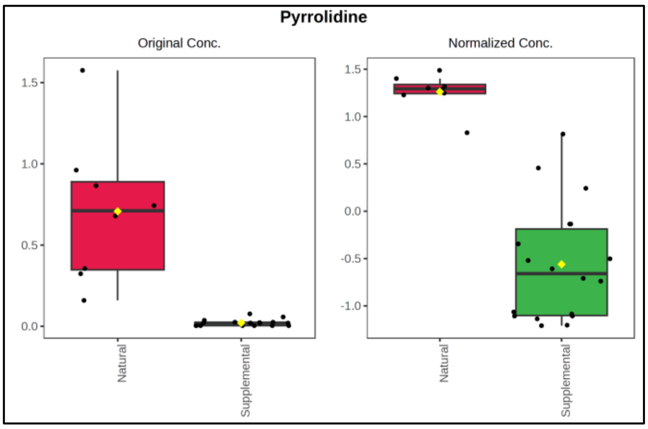
Pyrrolidine
Original Conc.
Normalized Conc.
Natural
Supplemental

(h)

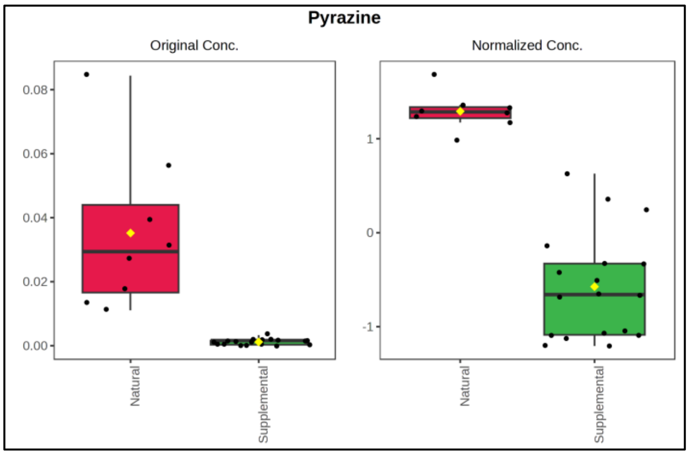
Pyrazine
Original Conc.
Normalized Conc.
Natural
Supplemental

(i)

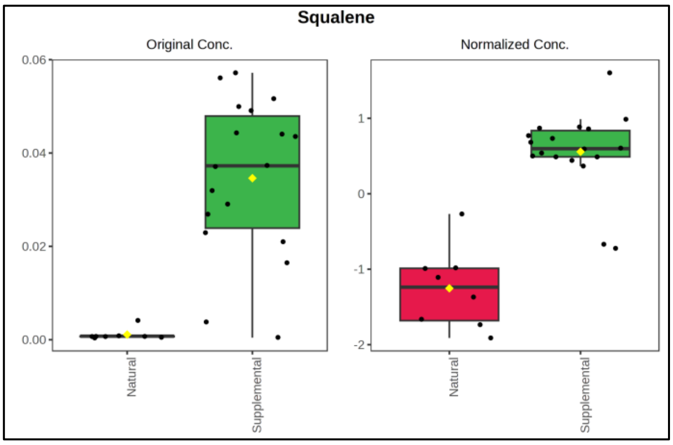
Squalene
Original Conc.
Normalized Conc.
Natural
Supplemental

(j)

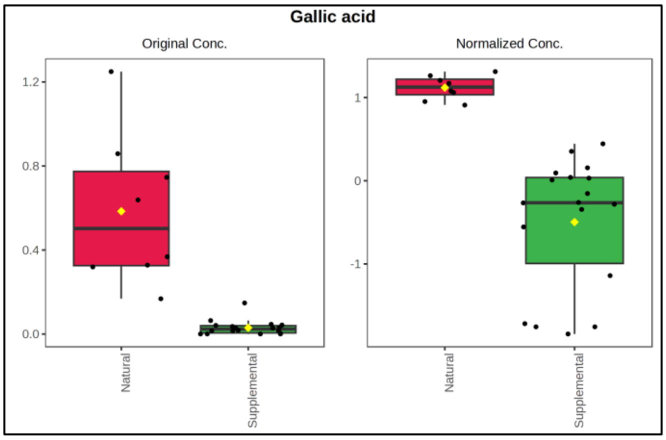
Gallic acid
Original Conc.
Normalized Conc.
Natural
Supplemental

(k)

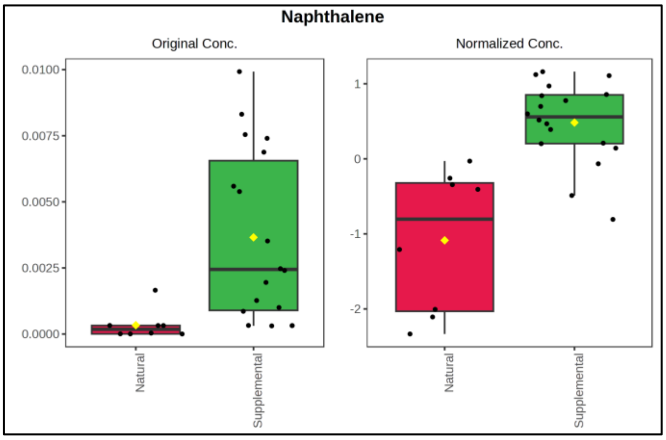
Naphthalene
Original Conc.
Normalized Conc.
Natural
Supplemental

(l)

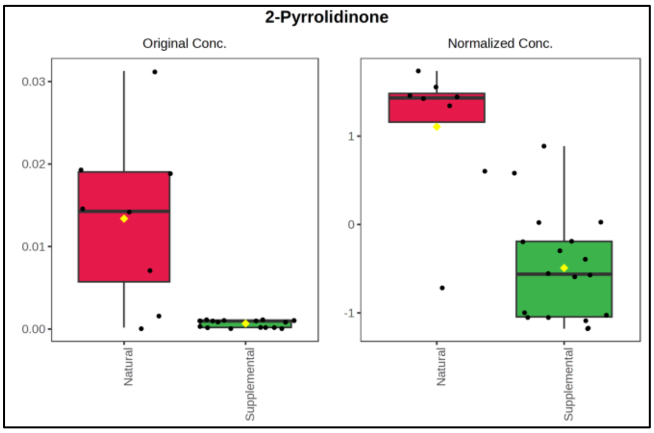
2-Pyrrolidinone
Original Conc.
Normalized Conc.
Natural
Supplemental

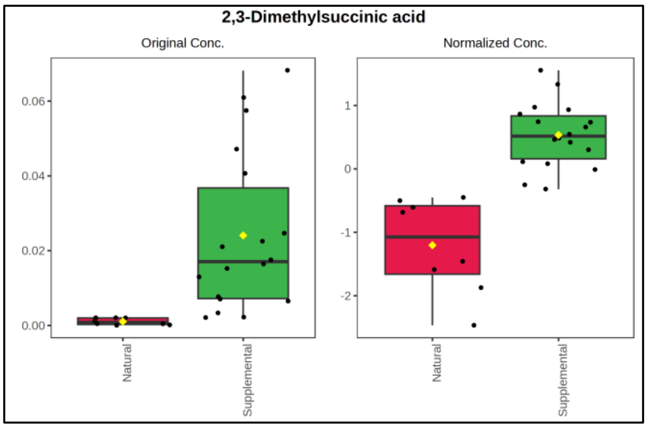
2,3-Dimethylsuccinic acid
Original Conc.
Normalized Conc.
Natural
Supplemental

(m)

(n)

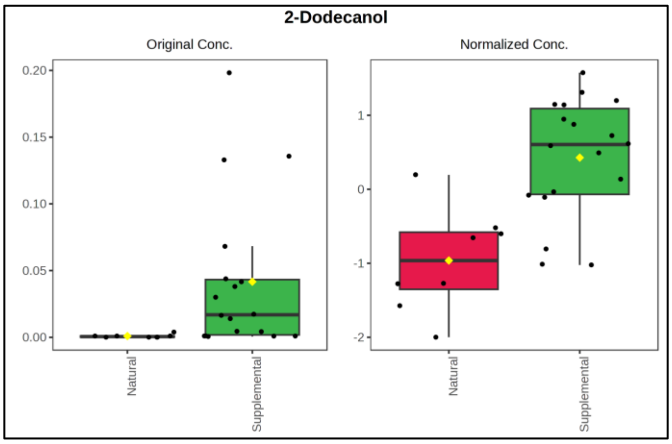
2-Dodecanol
Original Conc.
Normalized Conc.
Natural
Supplemental

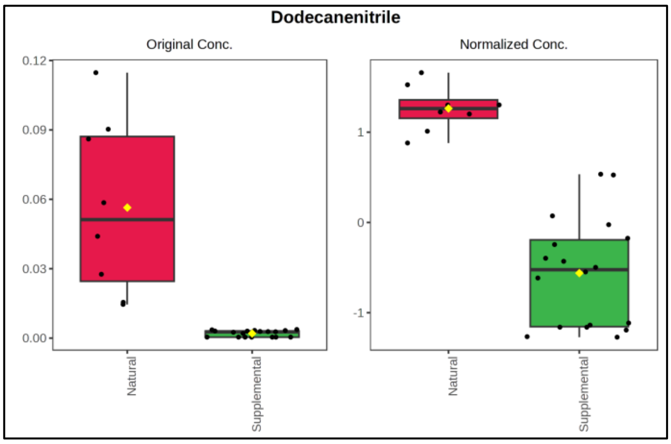
Dodecanenitrile
Original Conc.
Normalized Conc.
Natural
Supplemental

(o)

(p)

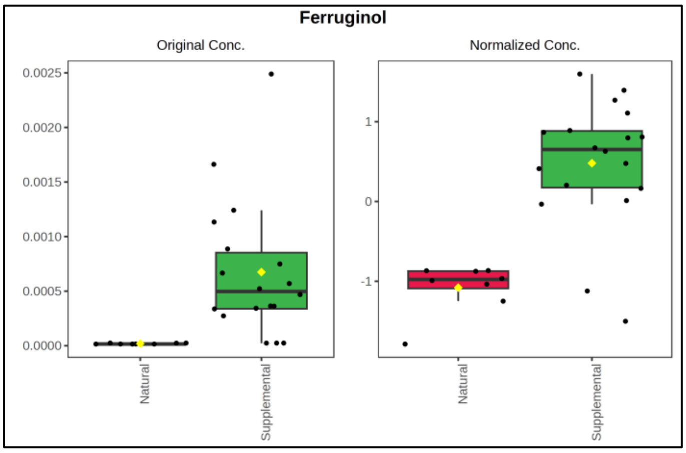
Ferruginol
Original Conc.
Normalized Conc.
Natural
Supplemental

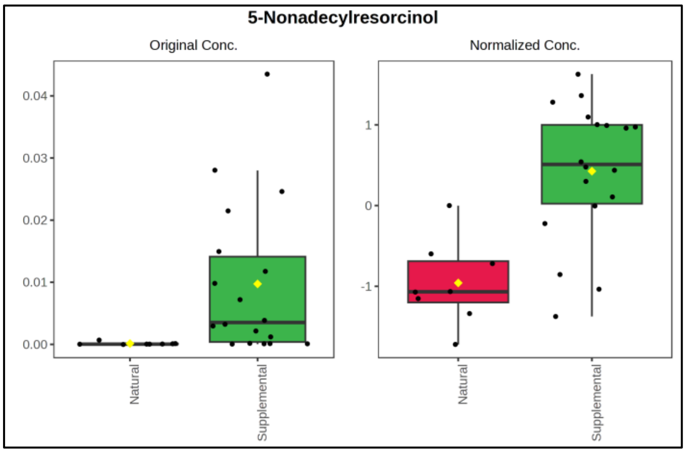
5-Nonadecylresorcinol
Original Conc.
Normalized Conc.
Natural
Supplemental

(q)

(r)

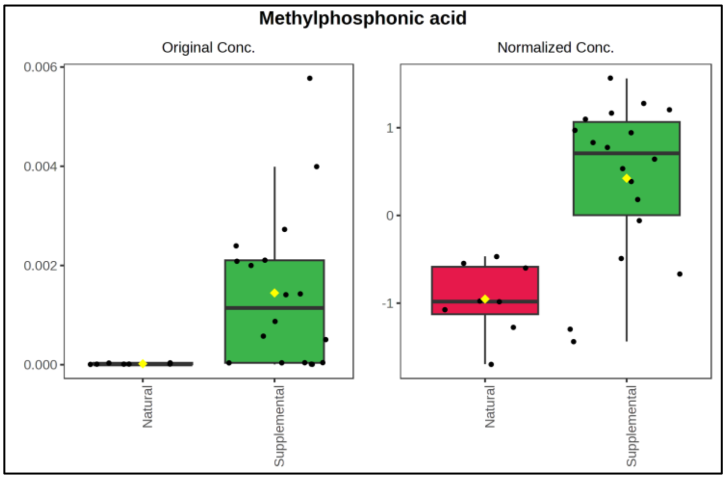

(s)

Figure S1: Significant ($p < 0.05$) metabolites with a fold change test (Log2 [FC]> 4) identified for giraffe receiving supplemental feed (SF) or only natural available vegetation (NAV): (**a**) Threonine; (**b**) 5-Oxoproline; (**c**) Serine; (**d**) Aspartic acid; (**e**) Alanine; (**f**) Glutamic acid; (**g**) 3-Hydroxyphenylacetic acid; (**h**) Pyrrolidine; (**i**) Pyrazine; (**j**) Squalene; (**k**) Gallic acid; (**l**) Naphthalene; (**m**) 2-Pyrrolidinone; (**n**) 2,3-Dimethylsuccinic acid; (**o**) 2-Dodecanol; (**p**) Dodecanenitrile; (**q**) Ferruginol; (**r**) 5-Nonadecylresorcinol; (**s**) Methylphosphonic acid.

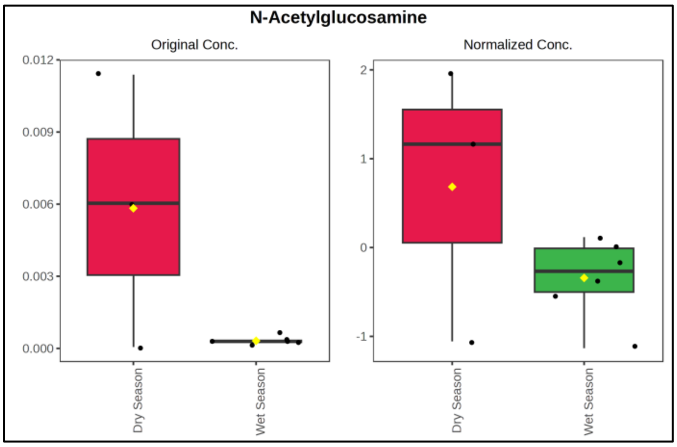
N-Acetylglucosamine
Original Conc.
Normalized Conc.
0.012
0.009
0.006
0.003
0.000
2
1
0
-1
Dry Season
Wet Season
Dry Season
Wet Season

(a)

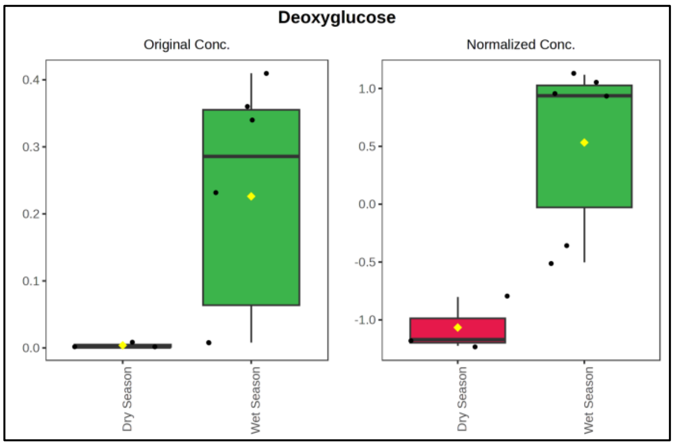
Deoxyglucose
Original Conc.
Normalized Conc.
0.4
0.3
0.2
0.1
0.0
1.0
0.5
0.0
-0.5
-1.0
Dry Season
Wet Season
Dry Season
Wet Season

(b)

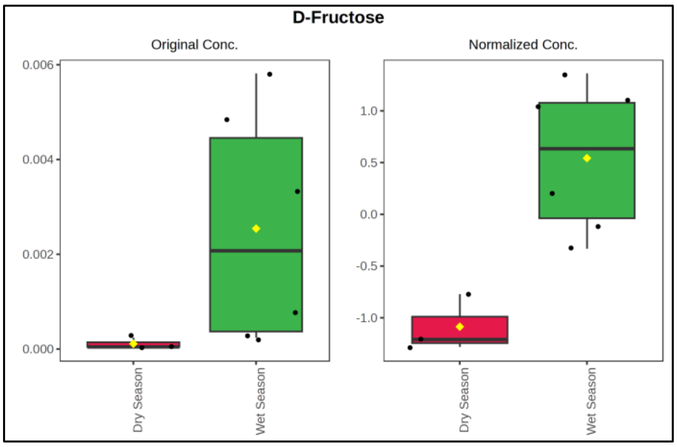
D-Fructose
Original Conc.
Normalized Conc.
0.006
0.004
0.002
0.000
1.0
0.5
0.0
-0.5
-1.0
Dry Season
Wet Season
Dry Season
Wet Season

(c)

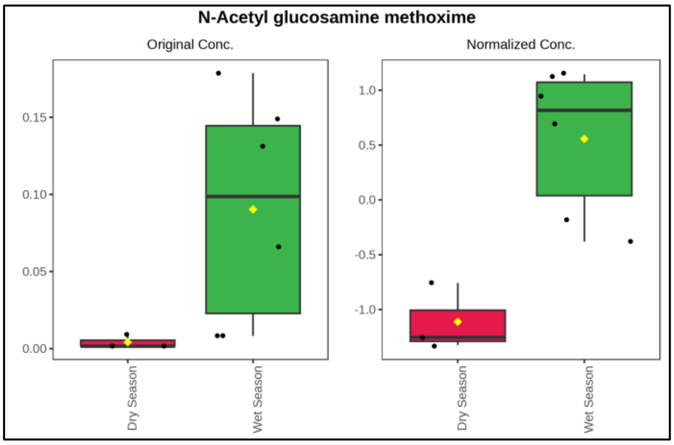
N-Acetyl glucosamine methoxime
Original Conc.
Normalized Conc.
0.15
0.10
0.05
0.00
1.0
0.5
0.0
-0.5
-1.0
Dry Season
Wet Season
Dry Season
Wet Season

(d)

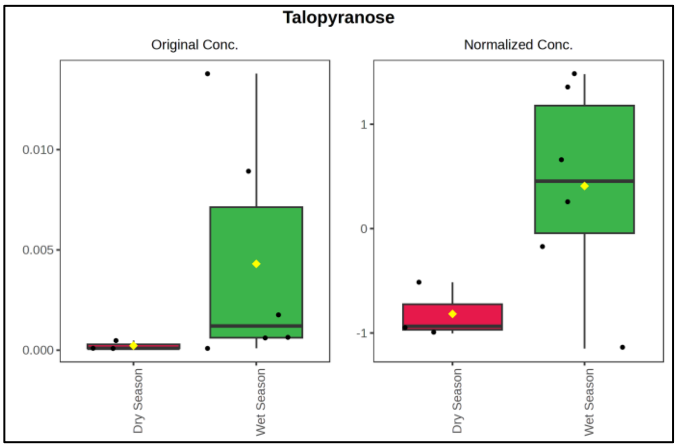
Talopyranose
Original Conc.
Normalized Conc.
0.010
0.005
0.000
1
0
-1
Dry Season
Wet Season
Dry Season
Wet Season

(e)

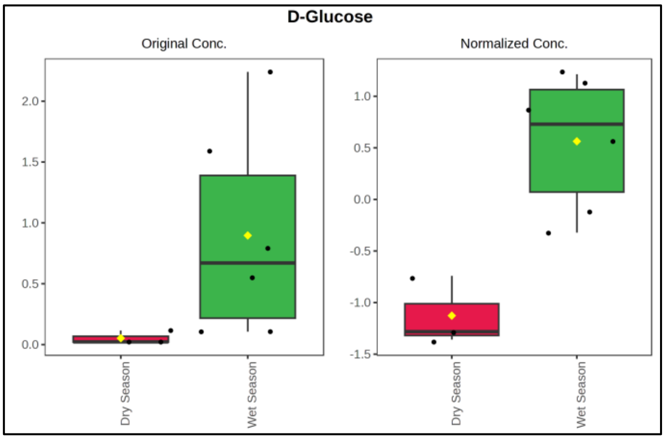
D-Glucose
Original Conc.
Normalized Conc.
2.0
1.5
1.0
0.5
0.0
1.0
0.5
0.0
-0.5
-1.0
-1.5
Dry Season
Wet Season
Dry Season
Wet Season

(f)

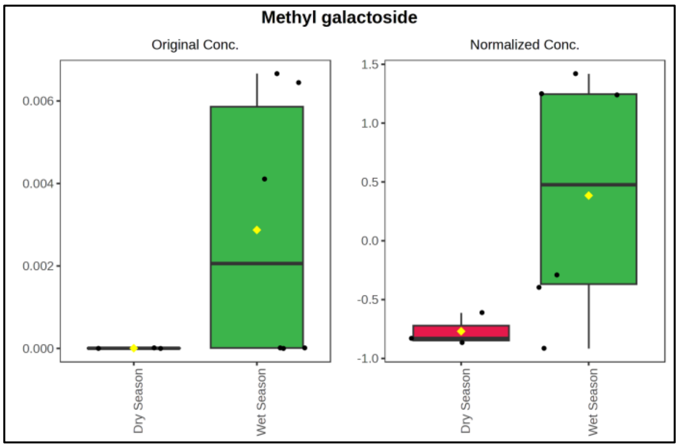
Methyl galactoside
Original Conc.
Normalized Conc.
Dry Season
Wet Season

(g)

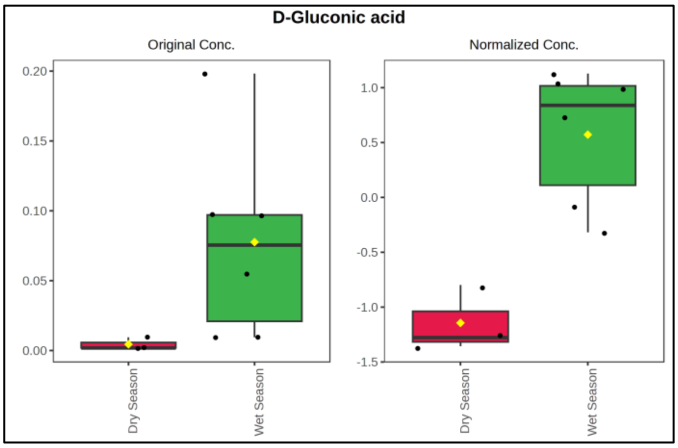
D-Gluconic acid
Original Conc.
Normalized Conc.
Dry Season
Wet Season

(h)

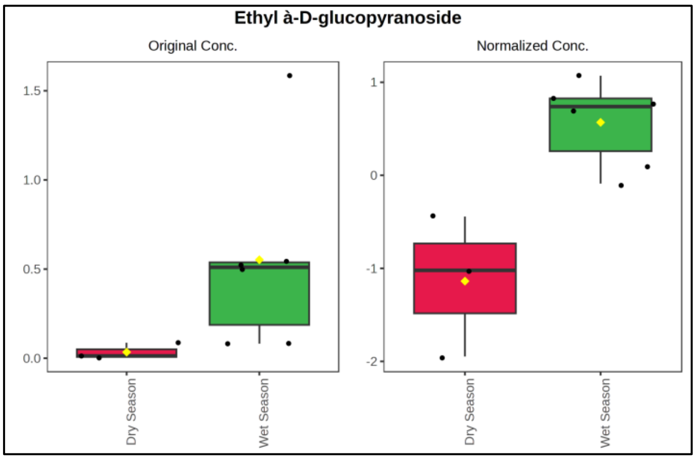
Ethyl à-D-glucopyranoside
Original Conc.
Normalized Conc.
Dry Season
Wet Season

(i)

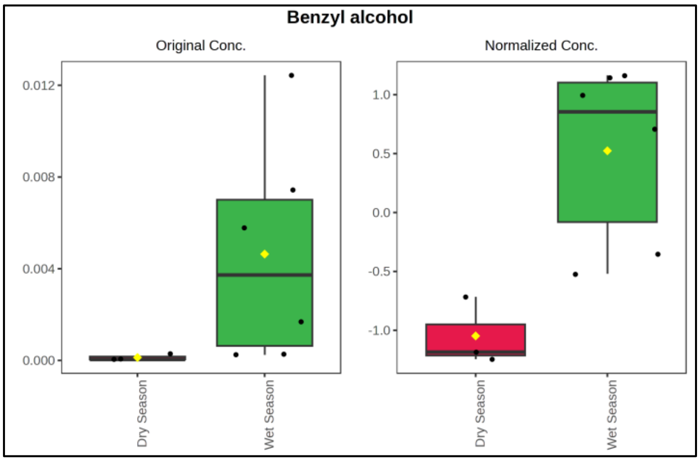
Benzyl alcohol
Original Conc.
Normalized Conc.
Dry Season
Wet Season

(j)

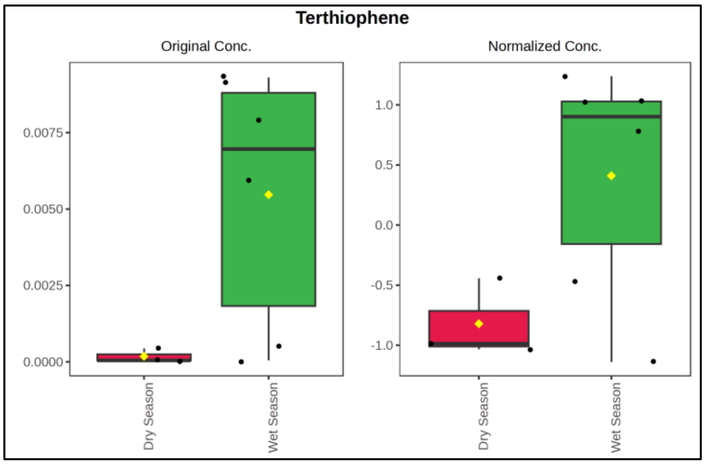
Terthiophene
Original Conc.
Normalized Conc.
Dry Season
Wet Season

(k)

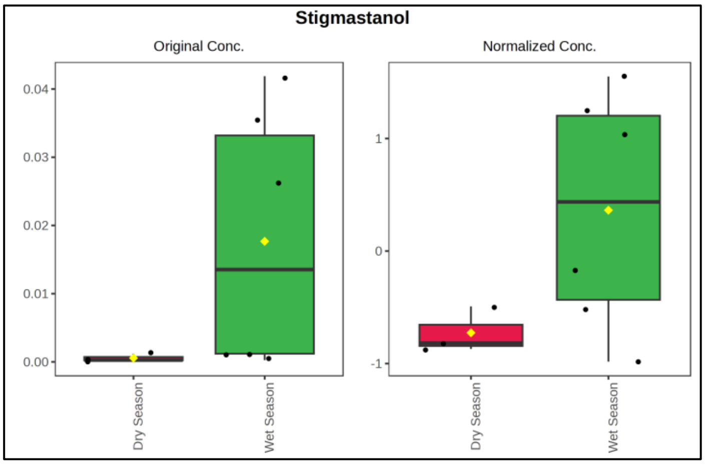
Stigmastanol
Original Conc.
Normalized Conc.
Dry Season
Wet Season

(l)

57

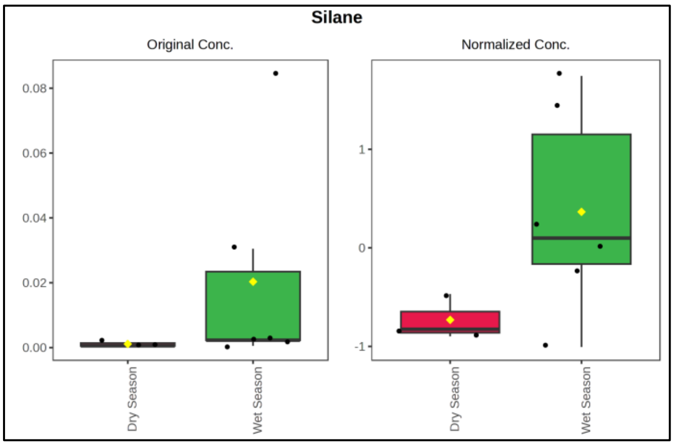

(m)

Figure S2: Significant ($p < 0.05$) metabolites with a fold change test (Log² [FC]> 4) identified for the wet and dry seasons from faeces 64
collected from a single adult male giraffe: (**a**) N-Acetylglucosamine; (**b**) Deoxyglucose; (**c**) D-Fructose; (**d**) N-Acetyl glucosamine 65
methoxime; (**e**) Talopyranose; (**f**) d-Glucose; (**g**) Methyl galactoside; (**h**) D-Gluconic acid; (**i**) Ethyl à-D-glucopyranoside; (**j**) Benzyl 66
alcohol; (**k**) Terthiophene; (**l**) Stigmastanol; (**m**) Silane. 67

metabolites MDPI

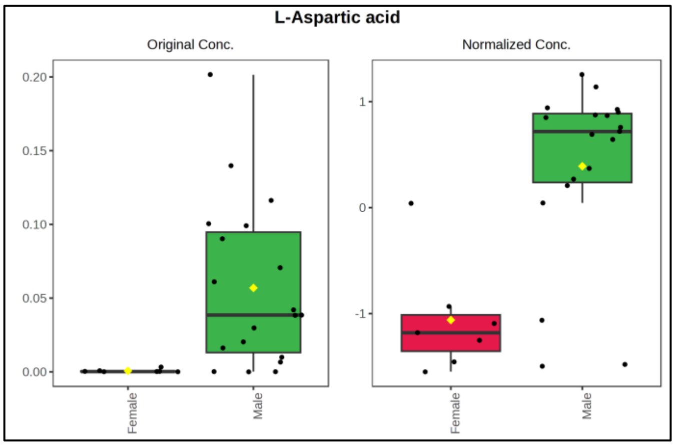

(a)

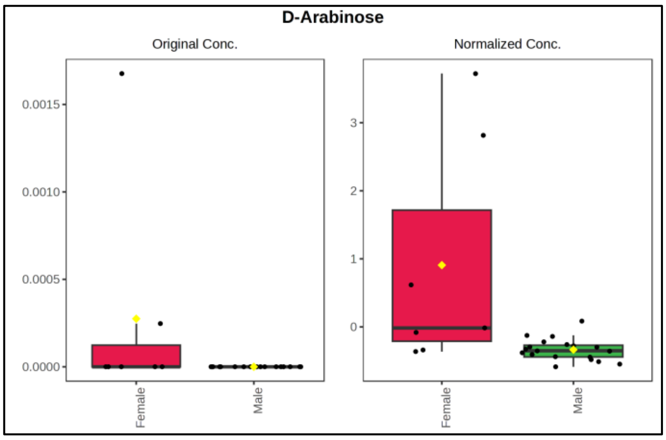

(b)

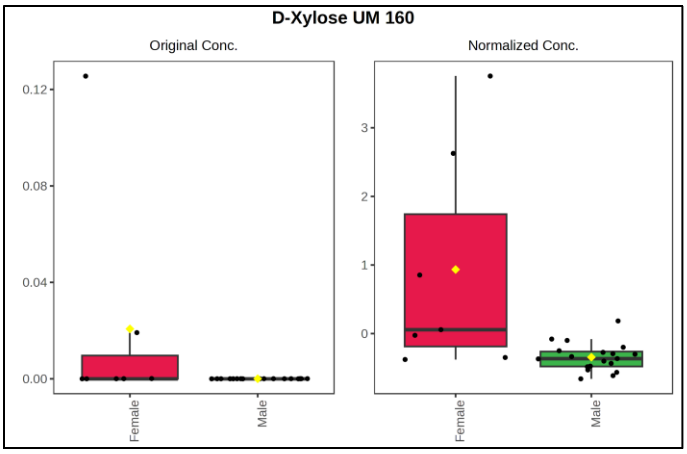

(c)

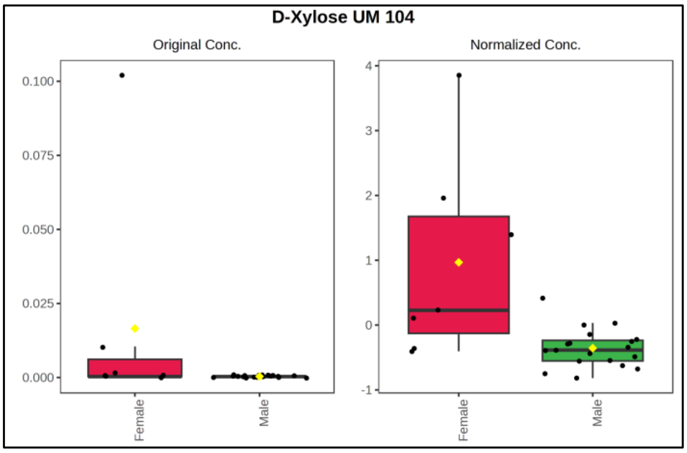

(d)

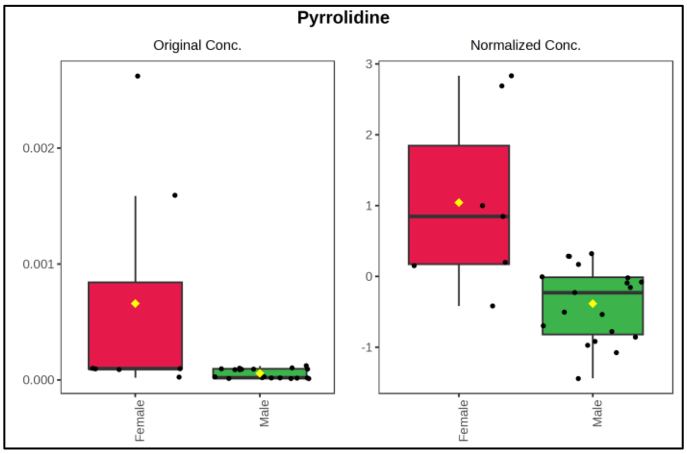

(e)

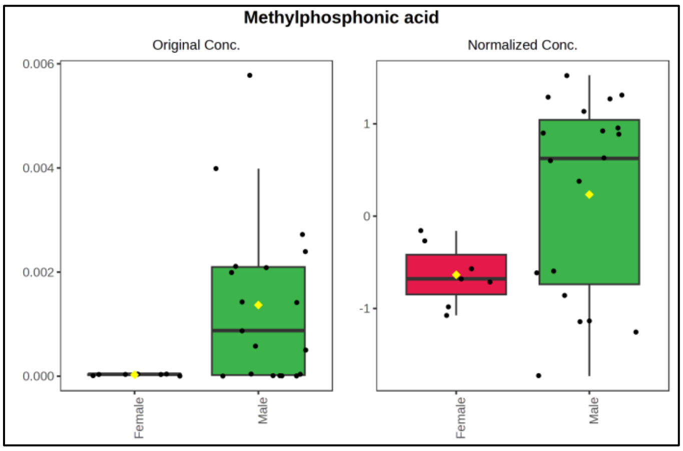

(f)

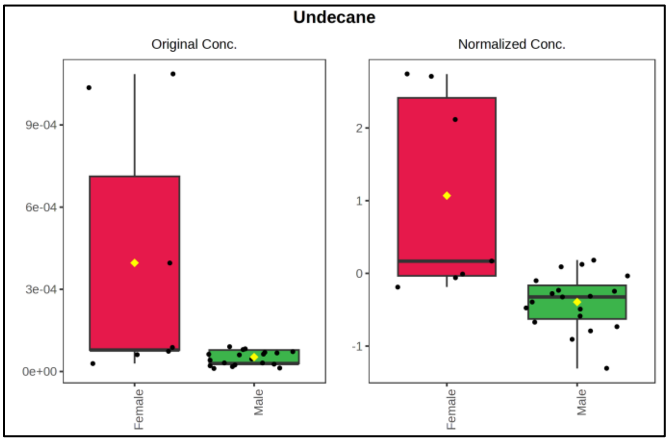

(g)

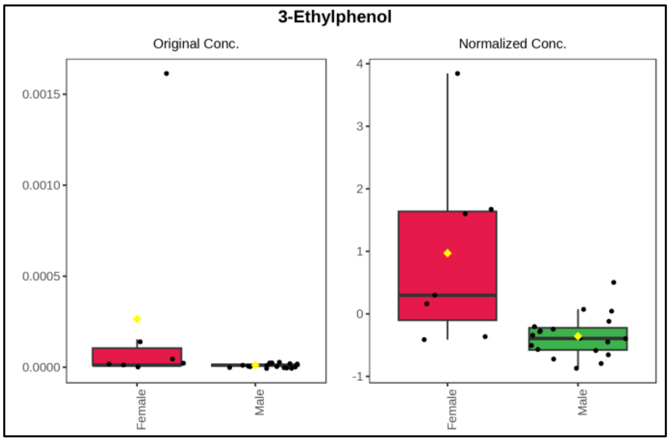

(h)

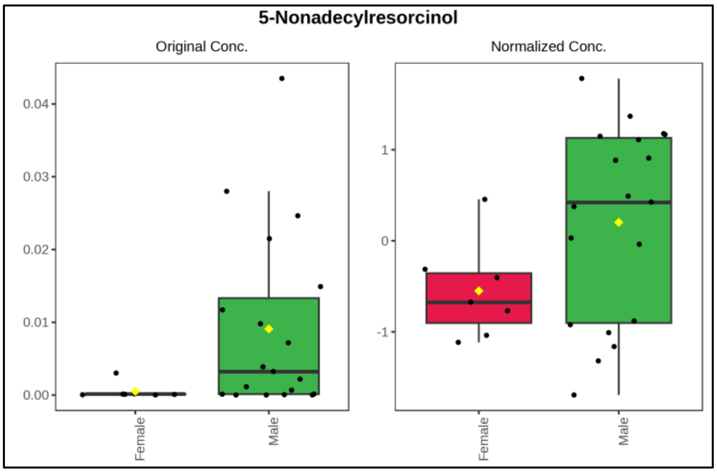

(i)

Figure S3: Significant ($p < 0.05$) metabolites with a fold change test (Log^2 [FC]> 4) identified for male and female giraffes: (**a**) L-Aspartic acid; (**b**) D-Arabinose; (**c**) D-Xylose UM 160; (**d**) D-Xylose UM 104; (**e**) Pyrrolidine; (**f**) Methylphosphonic acid; (**g**) Undecane; (**h**) 3-Ethylphenol; (**i**) 5-Nonadecylresorcinol.

Disclaimer/Publisher's Note: The statements, opinions and data contained in all publications are solely those of the individual author(s) and contributor(s) and not of MDPI and/or the editor(s). MDPI and/or the editor(s) disclaim responsibility for any injury to people or property resulting from any ideas, methods, instructions or products referred to in the content.
